# Supplementary material for: Determination of the trend of incidence of cutaneous leishmaniasis in Kerman province 2014-2020 and forecasting until 2023. A time series study
Source: PLoS Negl Trop Dis. 2022 Apr 11;16(4):e0010250. doi: 10.1371/journal.pntd.0010250 (PMC9049530; doi:10.1371/journal.pntd.0010250)
Supplement: Attachment — Submitted filename: response to reviewers.docx [file pntd.0010250.s002.docx]

Responses to reviewer's comments

--------------------
**Response to Reviewer #1comments:**

**Methods**

The study design, objectives and hypothesis are suitable and articulated. Aggregated data on cutaneous leishmaniasis incidence and meteorological information were used. The sample size is the cumulative number of cutaneous leishmaniasis cases between 2014 and 2020 in Kerman Province, Iran. The authors applied the correct statistical tests to support their conclusions. The authors describe that the study was approved by a local research ethics committee.

Thank you

**Results**

The analysis presented match the analysis plan. However, the results need to be completely presented. For example, Table 1 should present data by year and not only aggregated parameters for the entire period (2014-2020).

Thank you for your note. We modified the table1and presented data by year.

The legend in figure 2 needs to be improved. It is also necessary to assign letters to its three images. The legend in table 3 needs to be improved.

Thank you for your note. We suppose that figure 1, which simply shows the number of cases within the studied years doesn't need any legend. However, we added letters to each 3 images in figure3 and improved its title.

In figure 4, please indicate what are: black line, blue line, light and dark blue clouds.

Thank you for your note. We added legend to the figure 4.

**Conclusion**

The conclusions are supported by the data presented. But the authors need to clearly define the main limitations of the study.

Thank you for your note. We added the study limitations at the end of discussion.

The results need to be more widely discussed and compared to other endemic localities for cutaneous leishmaniasis.

Thank you for your note. We extensively revised the discussion and tried to improve it significantly by adding a wide range of studies and possible evidence based explanations for our findings.

**Editorial and Data Presentation Modifications**

The word leishmaniasis is misspelled several times throughout the text. Please correct.

Thank you for your note. Now it reads in correct way.

In lines 49-52, please use more recent references.

Thank you for your note. We added new references.

In lines 62-63, the authors indicate that "the burden of the disease has not decreased significantly in recent years". The authors' data show an opposite trend for the Kerman province.

Thank you for your note. It was corrected and highlighted in text.

**Summary and General Comments**

Afshar et al. evaluated the incidence of cutaneous leishmaniasis in Kerman province (Iran) from 2014 to 2020 and estimated its incidence until 2023. Study strengths: The research question is original, relevant to the field and of interest to the journal's readers. The method used is robust, providing reliable and quality results. Study weaknesses: the results need to be more widely discussed and compared to other endemic localities for cutaneous leishmaniasis.

Thank you for your note. We revised the discussion and tried to improve it significantly by adding a wide range of studies and possible evidence based explanations for our findings.

**Response to Reviewer #2comments:**

**Methods**

The manuscript is adequate in terms of its methodology. However, the exposition of the statistical methods needs improvement. The models being used should be described in more detail, at least up to the point that it is clear to the reader how to interpret the various model parameters that are mentioned.

Thank you for your note. Before going through the statistical tests, we added some explanation about the seasonality and main assumption of time-series analysis. We also explained about the meaning of auto correlation functions including ACF and PACF and interpretation of corresponding plots. We added more detailed information to the methods section. We also added some explanation about the graph axis at the end of figure3 title.

**Conclusions**

I think the interpretation and discussion of the results could be more extensive and insightful.

Thank you for your note. We revised the discussion and conclusion as well.

The authors should clarify how their result of a negative association between rainfall at a lag of 0 and incidence of cutaneous leishmaniasis compares with findings in the literature that appear to suggest the opposite. Maybe this has to do with the lag times under consideration in other studies? In any case, the authors should clarify.

Thank you for note. Based on your valuable comment, we extensively revised this part and tried to add some possible explanations as highlighted in the text.

**Editorial and Data Presentation Modifications**

The writing in the paper can and should be improved. Please see my comments for some suggestions, but I recommend the authors go through the entire paper carefully to ensure that the wording is not awkward or unclear.

**Summary and General Comments**

The manuscript needs improvement in several ways. I found the discussion and interpretation of the results to be rather brief, and a more thorough, insightful discussion would improve the manuscript.

Thank you for your note. We extensively revised the discussion and tried to improve it significantly by adding a wide range of studies and possible evidence based explanations for our findings.

Please see my comments regarding the potential confusion related to the association between rainfall and CL incidence.

Thank you for note. Based on your valuable comment, we extensively revised this part and tried to add some possible explanations as highlighted in the text.

Finally, the exposition of the statistical models that are used should be more detailed, and the writing in general can use some improvement.
 Thank you for note. We added more details to the method section make it more reader-friendly.

Here are some specific comments/recommendations:

line 36: include confidence intervals
Thank you for note It was corrected in text

line 36: instead of writing the variables in quotation marks, describe them properly
Thank you for note It was corrected in text

line 43: change "a method" to "methods"

Thank you for note It was corrected in text

lines 53-55: change wording. Perhaps "WHO reported that the highest rates of the disease were in 10 countries which accounted for 70-75% of cases: ..."

Thank you for note It was corrected in text

line 70: Why only predict up to the year 2023? And what does the study hope to achieve by predicting the trend for the next two years? This needs to be clarified to provide proper motivation.

Thank you for note. The reason is that we had time series data only on 7 consecutive years and in the absence of more data points, predicting the distant future couldn't be accurate enough. To obtain more accurate findings, we limited our prediction up to 2 upcoming years. Beside, our main goal was to study the disease trend over the time and depict its current situation to provide policy makers appropriate evidences for suitable actions.

line 73: Use commas and decimal points as appropriate in the numbers. Also, I think the population is more like 3.164718 million (it is missing a decimal point). Please correct.
Thank you for note It was corrected in text

line 74: It is a bit incomplete to note that it accounts for nearly 11 percent of the land area of Iran but not note what percent of the population it accounts for.
Thank you for note It was corrected in text

lines 80-81: change "obtained from vice chancellor of health" to "was obtained from the vice chancellor of health"

Thank you for note It was corrected in text

lines 90-96: Please provide more explanation about the statistical methods that you are using. Your explanation of the statistical models should be brief, but still detailed enough that the reader understands what the variables that you refer to correspond to.

Thank you for note It was corrected in text
line 131: Change "is also given" to "are also given"
Thank you for note It was corrected in text
line 178: Change wording of "an overall trend was decreasing" so that it is more clear.

Thank you for note It was corrected in text

line 179: Put comma after "As we showed"

Thank you for note It was corrected in text


lines 188-189: I am confused: if a reduction of rainfall is associated with a decrease in the incidence of CL, isn't that a positive association between rainfall and incidence? How does this compare to your findings of a negative association between rainfall at a lag of 0 and CL incidence? lines 204-205: Again, a comment about reduced rainfall contributing to a decreasing trend of CL incidence. How do you reconcile this with your inference of rainfall (at the lag of 0) being negatively associated with CL incidence?

Thank you for note. We extensively revised this part and tried to add some possible explanations as highlighted in the text.
